# A systematic review on EEG-based neuromarketing: recent trends and analyzing techniques

**DOI:** 10.1186/s40708-024-00229-8

**Published:** 2024-06-05

**Authors:** Md. Fazlul Karim Khondakar, Md. Hasib Sarowar, Mehdi Hasan Chowdhury, Sumit Majumder, Md. Azad Hossain, M. Ali Akber Dewan, Quazi Delwar Hossain

**Affiliations:** 1 Department of Biomedical Engineering, Chittagong University of Engineering & Technology, Chittagong, Bangladesh; 2 Department of Electrical & Electronic Engineering, Chittagong University of Engineering & Technology, Chittagong, Bangladesh; 3 Department of Electronics & Telecommunication Engineering, Chittagong University of Engineering & Technology, Chittagong, Bangladesh; 4 School of Computing and Information Systems, Faculty of Science and Technology, Athabasca University, Athabasca, AB T9S 3A3 Canada

**Keywords:** Neuromarketing, Brain-computer interface (BCI), Electroencephalography (EEG), Research trend, Pre-processing, Feature, Classification, Machine learning

## Abstract

Neuromarketing is an emerging research field that aims to understand consumers’ decision-making processes when choosing which product to buy. This information is highly sought after by businesses looking to improve their marketing strategies by understanding what leaves a positive or negative impression on consumers. It has the potential to revolutionize the marketing industry by enabling companies to offer engaging experiences, create more effective advertisements, avoid the wrong marketing strategies, and ultimately save millions of dollars for businesses. Therefore, good documentation is necessary to capture the current research situation in this vital sector. In this article, we present a systematic review of EEG-based Neuromarketing. We aim to shed light on the research trends, technical scopes, and potential opportunities in this field. We reviewed recent publications from valid databases and divided the popular research topics in Neuromarketing into five clusters to present the current research trend in this field. We also discuss the brain regions that are activated when making purchase decisions and their relevance to Neuromarketing applications. The article provides appropriate illustrations of marketing stimuli that can elicit authentic impressions from consumers' minds, the techniques used to process and analyze recorded brain data, and the current strategies employed to interpret the data. Finally, we offer recommendations to upcoming researchers to help them investigate the possibilities in this area more efficiently in the future.

## Introduction

Marketing is the process of introducing a product to the market. A good product cannot inform, engage, and sustain its target audiences without efficient marketing. Marketing serves as the conduit between a product and customers, influencing the final sale. Businesses find it challenging to expand and remain viable without receiving quantitative or qualitative feedback from their customers. In order to thrive in a highly competitive market, newly introduced products demand even more effective marketing strategies [1].

Traditional marketing largely relies on subjective focus-group assessments, interviews, and surveys. However, due to social desirability bias, consumers might not always convey their genuine thoughts [2]. They might not express their feelings in words but instead what they believe others would say in response [3]. People’s emotional states or surrounding circumstances at the time of self-reporting are also considered by these post-hoc analytic techniques [4]. Marketers and academics look for supplementary or alternative methods to address the limitations of traditional marketing strategies. One such option is to use Neuromarketing which analyzes customers’ spontaneous reactions to certain advertising campaigns, packaging, designs, etc. and explores how customers react to marketing stimuli.

The term “Neuromarketing” was first introduced by Ale Smidts in 2002 to refer to research on the application of neuroscience technology in the marketing sector [5]. It utilizes insights from neuroscience and cognitive science to precisely determine consumer requirements, desires, and preferences. It aids in creating marketing plans and campaigns that are appealing to the target market. By asking customers to respond to surveys after the fact, traditional research techniques concentrate mostly on the posterior attitude of consumers toward products. These replies don't accurately reflect the customer's natural state of mind at the moment of purchase since they are delayed and simplified [6]. By considering the brain signals at the moment of purchase, Neuromarketing, on the other hand, focuses on capturing the in-situ reaction.

Brain Computer Interface (BCI) is a cutting-edge technology that enables direct communication between the brain's electrical activity and an external device, typically a computer. Neuromarketing uses BCI technologies to gain insights into how consumers react to marketing stimuli. It is well recognized that a customer's decision-making process is influenced by various sophisticated aspects, and Neuromarketing offers profound insights into customer behaviors individually. Accordingly, Neuromarketing aids marketers in formulating plans by employing in-the-moment measurements of brain activity in response to diverse marketing stimuli. It uses a direct correlational process to explain consumer responses, in contrast to traditional approaches in marketing research. This objective approach enables marketers to create more successful and efficient tactics by better comprehending consumers' complicated and constantly changing mental processes. It should be noted that Neuromarketing is not necessarily aimed at influencing the specific consumer's personal decisions; instead, it seeks to improve the understanding of prospective customers' perspectives and interests to create precise behavioral models [7].

Neuromarketing studies how people's brains react to particular ads, package designs, products, etc., using non-invasive brain-scanning techniques, such as EEG [8–10], functional magnetic resonance imaging (fMRI) [11–13], functional near-infrared spectroscopy (fNIRS) [14, 15], etc. These neurophysiological data may now accurately represent customers' preferences, likes, and dislikes using modern feature extraction and classification algorithms [16]. Marketers utilize these results to produce advertising that customers find more attractive or encouraging. Among the neurophysiological signals, EEG has become the most popular and is widely used in the marketing sector owing to its low price and high temporal resolution. Additionally, because EEG signal changes cannot be consciously influenced, they serve as a more accurate objective measure of emotion [17]. EEG-based emotion recognition is a potential approach to understanding the mechanics behind emotional states and creating computational models for recognizing and forecasting consumer emotional responses. As a result, EEG-based techniques are frequently used in Neuromarketing to increase sales, advertising, package design, pricing, marketing campaigns, and other things. This review focused on the literature over the last seven years that used EEG data for analysis.

Large businesses like Google, Unilever, Microsoft, and others utilize the findings from more than 150 consumer neuroscientific firms that are commercially functioning worldwide to influence their consumers in a targeted and effective manner [1]. This innovation has been made possible by academic research, particularly the tremendous analytical reliability of the engineering sector of Neuromarketing. Therefore, it is essential to look into the foundations of Neuromarketing to assess its scopes and capabilities and to offer fresh insight into this area. There have been several review articles on the theoretical aspects of consumer neuroscience, covering various disciplines such as marketing, business ethics, management, psychology, and consumer behavior [18–28]. However, there is a lack of comprehensive engineering-focused review articles that specifically concentrate on the techniques for recording and processing brain activity and the methods used for interpreting the results in this field [1, 7, 29–32]. Rawnaque et al. [1] performed a systematic literature review from the engineering perspective, focusing on neural recording tools and interpretational methodologies used in this field. Khurana et al. [7] ran a literature survey focusing on Neuromarketing strategies, achievable information types, marketing stimuli, machine learning techniques for classification, and ethical implications. In another review, Pei and Li [29] discussed different EEG features, such as Event-Related Potentials (ERPs), EEG time–frequency components, and various classification techniques. Byrne et al. [30] discussed the EEG measures that can be used to predict customer preferences accurately. They have focused on the effects of theta-band power, ERP components, and machine-learning techniques for classification purposes. Hakim and Levy [31] explored the characteristics that have the potential to capture consumers' assessment process. These include inter-subject correlations (ISC), components acquired from an ERP design, hemispheric asymmetry, and various spectral band powers. Kalaganis et al. [32] discussed the hybrid EEG schemes used in Neuromarketing research, such as combining EEG with eye-tracking, electrodermal activity, heart rate, and other techniques. They also discussed the ethical issues related to these hybrid schemes.

**Fig. 1:**
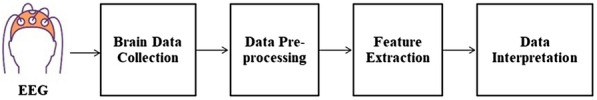
Typical workflow for Neuromarketing research

Researchers will be better supported with their research work and be able to move their research careers in the right direction if they keep up to date with the literature in their field of study and are proficient at doing so. It will benefit not only their professional development but also the development of the discipline as a whole. Therefore, knowing the most recent research trends will assist researchers in constructing their research framework properly. After setting the research objectives, a typical workflow for Neuromarketing research can be as presented in Fig. 1.

The process of collecting brain data from subjects to analyze their responses to different types of marketing stimuli is a complex one. Researchers attempt to create a realistic buying scenario to better understand the thoughts and preferences of consumers when selecting products. The brain data should be collected from the proper brain regions which effectively possess the emotions regarding the purchase intention. Once the data is collected, it is often very noisy and requires various pre-processing techniques to clean it up. There are many pre-processing techniques available, but the key is to effectively remove the noises while maintaining all necessary information. After the data has been pre-processed, necessary features are extracted from it. There are many feature extraction techniques available for EEG data extracting different time domain, frequency domain, and time–frequency domain features.

After extracting the features, they are utilized for classification purposes and statistical analyses. Various machine learning (ML) algorithms are used for preference classifications to predict the future preferences of consumers, while different statistical analyses are performed for various behavioral analyses. Proper selection of the processing techniques is essential to interpret the data better. Consequently, numerous technical stages must be completed for final data interpretation, which can prove to be challenging for future researchers to comprehend. In this sense, this article is set to answer the following questions:

- What is the current research trend in the field of Neuromarketing?
- What are the active brain regions that relate to the purchase intention of the consumers?
- What types of marketing stimuli should be used to elicit the genuine thoughts of the consumers?
- Which pre-processing techniques are suitable for EEG-based Neuromarketing applications?
- How different features relate to consumers’ decision-making process in EEG-based Neuromarketing applications?
- What are the techniques best suitable for interpreting the data in EEG-based Neuromarketing applications?

These questions will provide a thorough understanding of the most recent research scopes and techniques in consumer neuroscience. Following this short introduction, the methodology for this systematic review will be provided, followed by the key findings corresponding to the questions mentioned above and a discussion of the significant results.

## Methodology

**Fig. 2:**
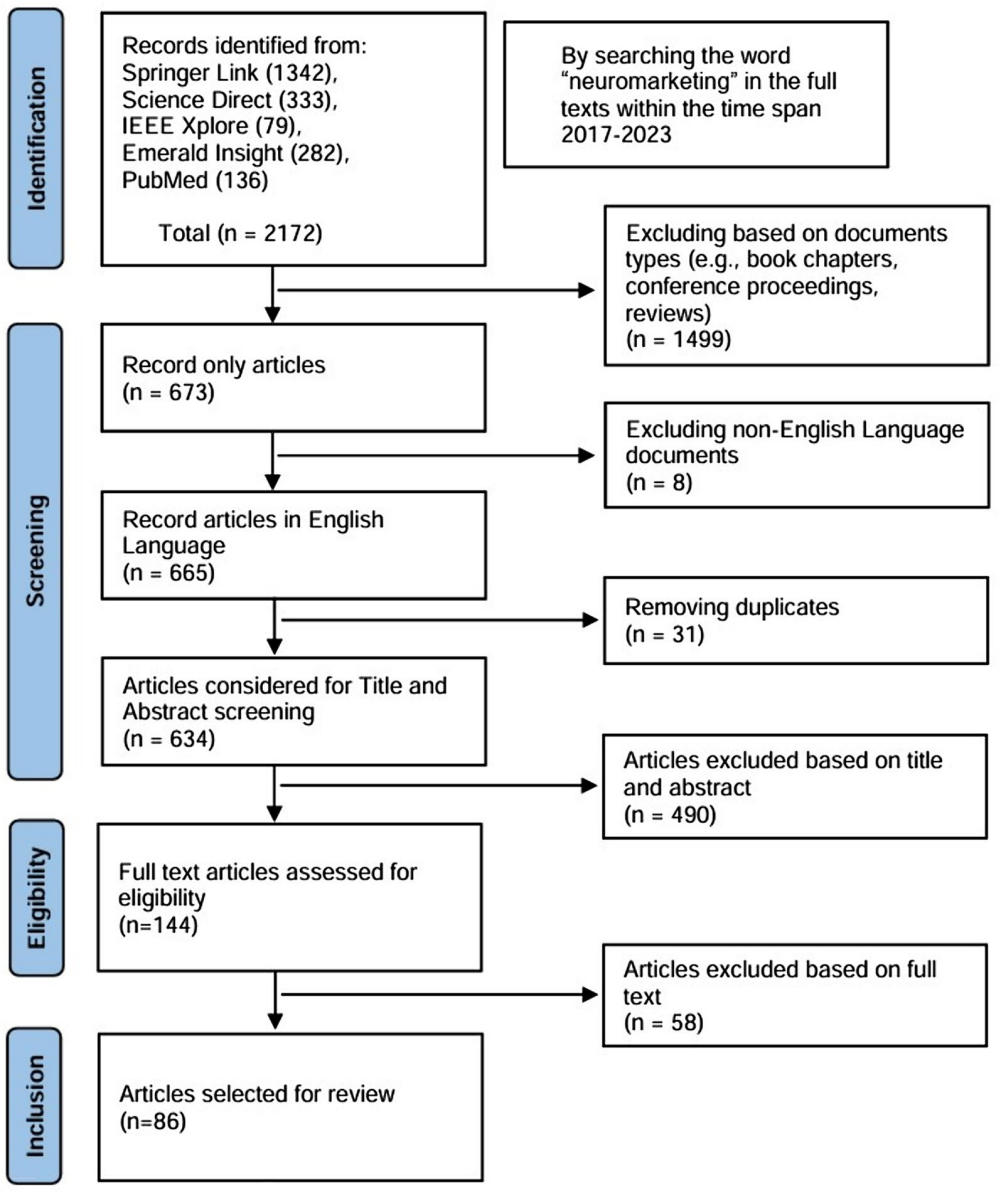
PRISMA flow chart for selecting publications for the systematic review

The systematic reviews are based on clearly stated questions, select relevant studies, evaluate their quality, and summarize the evidence using specific methods. They are distinguished from traditional reviews and commentary by their clear and systematic approach. A precise definition of the research question and a discussion of the inclusion–exclusion criteria are required for systematic reviews to determine the scope of the study. After a thorough review of the literature, papers should be chosen according to their relevance, and the findings of the selected research should be critically synthesized and evaluated to reach definite conclusions [33]. For this systematic review, we followed the instructions provided by the Preferred Reporting Items for Systematic Reviews and Meta-Analyses (PRISMA) to select original research articles [34]. The PRISMA protocol consists of four stages, as shown in Fig. 2—identification, screening, eligibility, and inclusion.

## Systematic review

The goal of consumer neuroscience is to establish physiological neurosis for understanding consumer behavior by combining neuroscientific techniques and theories with behavioral theories, validated models and designs from consumer psychology, and allied fields like behavioral decision sciences [35]. In this journey of consumer neuroscience, much literature has been published to contribute to understanding consumer behavioral traits. This systematic review has reviewed the works done in the last seven years. This section will explore current Neuromarketing research trends and address the review's research questions.

### The research trend in neuromarketing

Being a relatively new field of research, Neuromarketing has drawn the attention of researchers in recent years. Many studies have been done in this field, and the research trend is evolving. Hence, upcoming researchers should pay attention to the trend. Here, we have performed a cluster analysis based on the research objectives of the previous studies to get to know the current research trend in Neuromarketing.

Early studies of this field used to focus on building the theoretical base of Neuromarketing, such as using the concept of Neuroscience in marketing, economics, and customer behavior, and also to predict the preference of consumers merely. But modern studies focused on building marketing strategies utilizing Neuroscientific methods. To comprehend the context of consumer decision-making and behavior, they explored the elements influencing consumer decisions from a multidisciplinary point of view. These studies looked at how brain responses can predict consumer behavior in relation to subtle changes in marketing aspects, such as packaging design, advertising message, pricing, and brand identification other than the product.

These research aspects can be used to form different groups so that cluster analysis can be performed to track the research trend in Neuromarketing. Such cluster analysis was conducted in the study by Shahriari et al. [21]. They reviewed the research articles from valid databases between 2005 and 2017 and divided them into 6 clusters with text-mining methodology. In this study, we have considered the research articles from valid databases between 2017 to 2023 and divided them into similar 5 clusters based on the research goals of the studies. One of the clusters from Shahriari et al. [21], namely Ethical Issues, has not been considered here since this review avoided theoretical/conceptual studies and considered only the research articles that directly contribute to the Neuromarketing research’s experimental findings. The names of the five clusters remain the same as in Shahriari et al. [21] to compare results between 2005–2017 and 2017–2023 and represent current research trends in Neuromarketing.

#### Cluster 1—ads and video commercials

The articles that deal with identifying emotion through brain data using different stimuli, such as e-commerce product images [8, 36], video ads [37, 38], product shapes [39] etc., have been considered under this cluster. These articles mainly focused on EEG-based emotion identification/preference prediction by adopting different ML techniques to train the models to predict the future preferences of the consumers. Yadava et al. [8] proposed a predictive model to catch consumers’ intentions toward E-commerce products. Teo et al. [39] investigated several deep learning (DL) architecture tunings for increasing the classification rate of the preference classification task. Aldayel et al. [40] used several feature sets of EEG indices to explore preference prediction. Oikonomou et al. [41] proposed a sparse classification scheme for recognizing cognitive and affective brain mechanism in Neuromarketing. In this review, about 22.09% (19 articles) of the total research articles have been found under this cluster as compared to 25% of Shahriari et al. [21] as presented in Table 1.

**Table 1:** The articles under the five clusters

Cluster	Neuromarketing studies (2017–23)	Percentage of studies (2005–17)	Percentage of studies (2017–23)
Ads and video commercials	Yadava et al. [8], Hakim et al. [36], Hakim et al. [37], Guixeres et al. [38], Teo et al. [39], Aldayel et al. [40], Oikonomou et al. [41], Aldayel et al. [42], Kumar et al. [9], Li et al. [43], Yen and Chiang [44], Zeng et al. [10], Raiesdana and Mousakhani [45], Kislov et al. [46], Al-Nafjan [47], Shah et al. [48], Hassani et al. [49], Georgiadis et al. [50], Göker [51]	25%	22.09%
Neuroscience in marketing, economics, and consumer behavior	Shen et al. [52], Uhm et al. [53], González-Morales [54], Wajid et al. [55], Gountas et al. [56], Domracheva and Kulikova [57], Wei et al. [58], Ramsøy et al. [59], Yang et al. [60], Harris et al. [61], Goto et al. [62], Eijlers et al. [63], Vozzi et al. [64], Zito et al. [65], Wang et al. [66], Ma et al. [67], Kakaria et al. [68], Lukovics et al. [69]	17%	20.93%
Marketing strategies	Goto et al. [70], Jin et al. [71], Çakar et al. [72], Royo et al. [73], Gong et al. [74], Daugherty et al. [75], Gholami Doborjeh et al. [76], Alonso Dos Santos and Calabuig Moreno [77], Gordon et al. [78], García-Madariaga et al. [79], Fu et al. [80], Golnar-Nik et al. [81], Sänger [82], Alvino et al. [83], Verhulst et al. [84], Zubair et al. [85], Hsu and Chen [86], Hsu and Chen [87], Pagan et al. [88], Zhao and Wang [89], Izadi et al. [90], Robertson et al. [91], Martinez-Levy et al. [92], Wang et al. [93], Ma et al. [94], Yu et al. [95], Mengual-Recuerda et al. [96], Kim et al. [97], Wang et al. [98], Alvino et al. [99], Russo et al. [100], Wang et al. [101], Hassani et al. [102], Wei et al. [103], Damião de Paula et al. [104], Russo et al. [105], Bosshard and Walla [106], Song et al. [107]	32%	44.19%
Advertising message components	Avinash et al. [108], Michael et al. [109], Hsu and Chen [110], Pennanen et al. [111], Leeuwis et al. [112], Uhm et al. [113]	6%	6.98%
Decision making process and brand selection	Ma et al. [114], Garczarek-Bąk et al. [115], Özbeyaz [116], Yang and Kim [117], Camarrone and Van Hulle [118]	10%	5.81%

#### Cluster 2—Neuroscience in marketing, economics, and consumer behavior

The research works that used the concept of Neuroscience in marketing, economics, and customer behavior, have been incorporated into this cluster. These articles mainly focused on building the theoretical base of Neuromarketing. Such studies were motivated to find out the fundamental neural basis and psychological processing working behind the customer decision-making mechanism [52, 53]. Some studies also investigated brain activation patterns while consumers choose products to buy [54]. From such neuropsychological perspectives, Kakaria et al. [68] compared cognitive load during planned and unplanned virtual shopping and Lukovics et al. [69] investigated customer acceptance of self-driving technology. We have found about 20.93% (18 articles) of the total research articles under this cluster as compared to 17% of Shahriari et al. [21] as presented in Table 1.

#### Cluster 3—Marketing strategies

The articles under this cluster investigated the elements influencing consumer decisions in relation to subtle changes in marketing aspects, such as packaging design, advertising message, languages used for ads, and pricing other than the product. These articles mainly focused on building marketing strategies utilizing Neuroscientific methods. Royo et al. [73] investigated whether product design and follow-up advertising and marketing using a continuous narrative approach in conjunction with verbal narrative advertising could provide more positive emotional responses from future product users than using visual narrative advertising. Alonso Dos Santos and Calabuig Moreno [77] presented a pilot study that sought to examine the focus on sponsor variables by evaluating the level of consent associated with sponsors and sponsored organizations. Gordon et al. [78] studied consumer reactions to record-based videos in energy-efficient social marketing. García-Madariaga et al. [79] explored consumers’ attention and preferences for three packaging attributes, image, text, and color, as separate variables. Fu et al. [80] investigated the impact of price deception on the consumer buying behavior and the neural mechanisms that underpin them. Golnar-Nik et al. [81] investigated the possibility of EEG spectral power for predicting customer preferences and interpreting changes in consumer buying behavior when the content of an advertisement, such as the backdrop color and promotions, was modified. Alvino et al. [83] assessed whether the EEG makes a significant and tangible contribution to predicting consumer behavior and preferences when using similar products of different prices. Hsu and Chen [86] looked into how hotel videos featuring a happy face emoji as a subtle subtext impact people’s hotel choice. Izadi et al. [90] looked at the neuropsychological reactions of customers to promotional strategies and their choice to purchase sports goods to establish the most successful method. Hassani et al. [102] investigated the impacts of products’ colors on consumers’ purchase decisions. The main objective of these studies was to investigate how subtle changes in marketing utilities affect consumers’ purchase behavior. We have found out about 44.19% (38 articles) of the total research articles under this cluster as compared to 32% of Shahriari et al. [21] as presented in Table 1.

#### Cluster 4—Advertising message components

Articles in this cluster discuss the emotional effects that various advertising message components have on consumers. The effects of music and visual stimuli were the main concerns of these articles. Avinash et al. [108] proposed a model to provide an eminent way to understand customer behavior through Neuromarketing auditory stimuli as advertisement jingles so that any company can launch the best advertisements jingle for promoting their business. Michael et al. [109] studied unconscious emotional and cognitive responses using travel images to understand the specific mental processes of travel behavior. Hsu and Chen [110] analyzed the aphrodisiac effects of musical stimuli during wine tasting. Uhm et al. [113] investigated the impact of music on viewers' responses to sports ads. In this systematic review, about 6.98% (6 articles) of the total research articles have been found under this cluster as compared to 6% of Shahriari et al. [21] as presented in Table 1.

#### Cluster 5—Decision making process and brand selection

The research studies under this cluster dealt with the effects of brand familiarity on consumers’ decision-making process. Ma et al. [114] examined brain characteristics impacted by brand image and product group in a brand portfolio experiment. Garczarek-Bąk et al. [115] explored the potential of forecasting brand sales based on psychophysiological responses to a retailer’s television commercial. Özbeyaz [116] studied consumer judgments for branded and unbranded stimuli using machine learning algorithms. In this study, about 5.81% (5 articles) of the total research articles have been found under this cluster as compared to 10% of Shahriari et al. [21] as presented in Table 1.

**Fig. 3:**
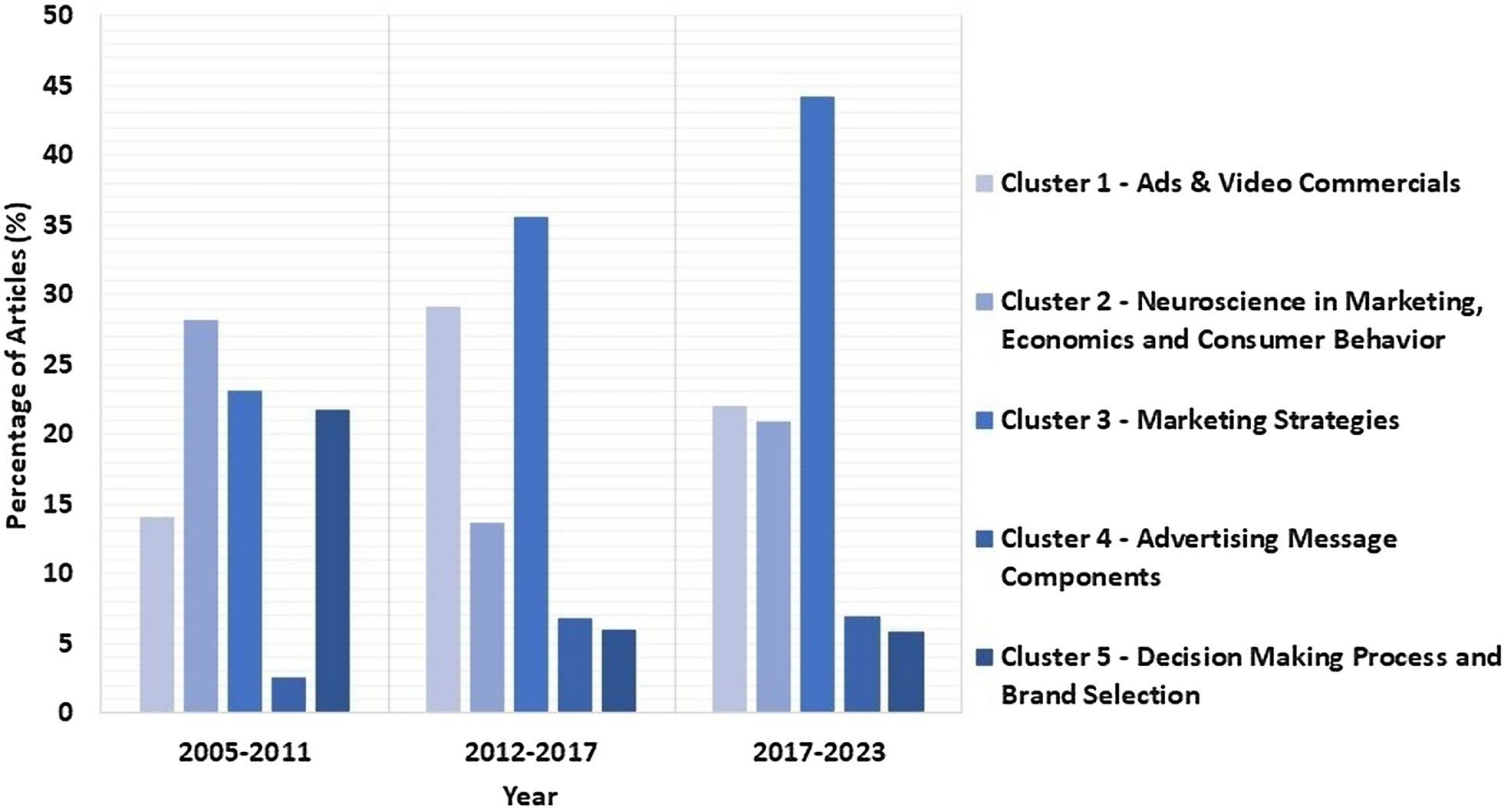
The research trend in Neuromarketing

According to the results of this study and the research of Shahriari et al. [21], the most popular topics in Neuromarketing from 2005 to 2023 are shown in Fig. 3. The data reveals that the number of articles on building marketing strategies has significantly increased over time, while the number of articles on other major topics has decreased. This indicates that the research trend in Neuromarketing has shifted from mere preference prediction of consumers to building marketing strategies using Neuroscience. Nevertheless, comparable research is ongoing for preference prediction in cluster 1 since understanding consumer preferences is one of the primary objectives of Neuromarketing. However, there is still no definitive method for predicting consumer preferences in Neuromarketing. With the advancements of artificial intelligence (AI) and ML techniques, researchers are increasingly interested in exploring this field of research. Another significant field is cluster 2, which aims to establish the theoretical basis of Neuromarketing. As a relatively new field of research, Neuromarketing enables researchers to develop principles for using Neuroscience concepts in marketing and economics to ensure its success.

Staying current with the research trend in the field of study supports researchers' work and career advancement, benefiting both professional development and the discipline as a whole, as it aids in creating a proper research framework. It is evident from the cluster analysis that even though some studies were found to be using concepts and principles of Neuromarketing to build up a theoretical base or to predict the purchase decision of consumers in the early years of Neuromarketing research, most studies nowadays utilized Neuroscience to create marketing strategies so that businesses become successful. Before setting the research goals, future Neuromarketing researchers should follow the research trend away from simple consumer preference prediction and toward developing marketing strategies incorporating Neuroscience.

Although Neuromarketing has many advantages in understanding human behaviour in various contexts, one of the biggest challenges relates to ethical concerns like maintaining privacy and confidentiality, safeguarding vulnerable groups, and providing an authentic interpretation of research findings. Therefore, researchers should focus their study on the top issues of recent years and look into the possibilities of integrating neuroscience into other marketing disciplines.

### Active brain regions in neuromarketing applications

The comprehension of the human brain's structure has shown to be essential in Neuromarketing research due to the close correlation between its functionality and the interpretation of neural responses [1]. The frontal, parietal, temporal, and occipital lobes are the four lobes that make up the complex outer layer of the human brain. Each lobe has a unique function related to motor, emotional, and cognitive responses. The frontal lobe is responsible for most of our conscious reasoning and decision-making [119]. The posterior section of the frontal lobe is in charge of movement-related decisions, while the prefrontal region handles cognitive decision-making. The left frontal area plays a role in the experience of positive emotions such as joy, interest, and happiness. These emotions encourage and sustain approach motivations. On the other hand, the right frontal area is responsible for negative emotions such as fear, disgust, and sadness. These emotions support and maintain withdrawal motivations [120]. The parietal lobe processes information about taste, touch, and movement. The temporal lobe is responsible for acoustic identification, visual memory, and integrating new sensory data with old memories. Finally, the occipital brain is the primary center for visual processing [1].

The EEG is a commonly used instrument to measure brain activity. It records the electrical activity on the scalp by analyzing the voltage differences produced by firing neurons in the brain. The EEG data acquisition system utilizes the international 10–20 method to place multiple electrodes directly on the head. This method provides information about the space between electrodes, specifically within 10–20% of the scalp boundaries from front to back or left to right. The distance between adjacent electrodes is 10% or 20% of the scalp diameter. The 10–20 standard has been commonly used in various EEG systems to enhance signal reliability and minimize signal-to-noise ratio. It is necessary to position the electrodes exactly above the relevant brain region in order to record the neuronal activity associated with a particular function.

The EEG activity can be divided into different frequency bands, such as delta (δ), theta (θ), alpha (α), beta (β), and gamma (γ). These frequency bands have a connection to emotional reactions. The theta band, located at the center of the frontal brain, shows how emotions are processed when a consumer views a product. The alpha band on the prefrontal cortex distinguishes between positive and negative emotional responses. The beta band is linked to changes in emotional arousal, while the gamma band is mainly related to the effects of arousal [121]. The brain activation is positively correlated with the beta band and negatively correlated with the alpha band. Approach motivation towards a product is indicated by higher beta and lower alpha in the left frontal than in the right frontal area. This difference in activity between the left and right frontal sides is called frontal asymmetry [65, 100, 120, 122]. Greater activation in the left prefrontal region, as indicated by higher values in the gamma band, can be correlated with consumers’ willingness to pay [59]. The activity level of the frontal theta in the prefrontal cortex indicates the cognitive processing that arises from mental exhaustion. Higher levels of theta activity are associated with more challenging and complicated tasks in the frontal area [123]. The mostly utilized EEG sub-bands along with their frequency ranges, brain regions of interest, and interpretations in Neuromarketing are summarized in Table 2.

**Table 2:** The EEG sub-bands’ interpretation in Neuromarketing

EEG band	Brain region and interpretation	Neuromarketing studies
Theta (θ) (4–8 Hz)	Fronto-central: Positively correlated with more challenging and complicated tasks	Golnar-Nik et al. [81], Avinash et al. [108], Modica et al. [123]
Alpha (α) (8–13 Hz)	Pre-frontal: Negatively correlated with brain activation—higher alpha in the right frontal area indicates approach motivation and vice versa	Zeng et al. [10], Al-Nafjan [47], Ramsøy et al. [59], Zito et al. [65], Martinez-Levy et al. [92], Russo et al. [100], Touchette and Lee [120], Al-Nafjan et al. [124]
Beta (β) (13–30 Hz)	Frontal: Positively correlated with brain activation—higher alpha in the left frontal area indicates approach motivation and vice versa	Zeng et al. [10], Al-Nafjan [47], Ramsøy et al. [59], Zito et al. [65], Martinez-Levy et al. [92], Russo et al. [100], Touchette and Lee [120], Al-Nafjan et al. [124]
Gamma (γ) (30 ~ Hz)	Pre-frontal: Positively correlated with brain activation—higher gamma in the left frontal area indicates more willingness-to-pay and vice versa	Zeng et al. [10], Al-Nafjan [47], Ramsøy et al. [59]

Understanding the structure of the human brain is crucial in neuromarketing research. It is closely linked to interpreting neural responses, making it necessary to position electrodes accurately over the relevant brain region to record the neuronal activity associated with a particular function. Although many researchers have found that the medial-frontal brain region is responsible for the preference function, there is still a need for more agreement on which electrodes should be used within the same brain area. Therefore, scanning a larger brain area focusing on the frontal region would be better.

### Stimuli in neuromarketing applications

To elucidate the decision-making process of consumers, different types of marketing stimuli have been used in the literature over the years, such as direct products, images of the products, video advertisements, normal pictures, affective images, music, movies, etc. These stimuli can be classified into three major categories, *Product, Promotion,* and *Others.* Here, we have categorized the previous studies based on the stimuli they used for data collection.

#### Product

Actual products and images of the products are considered in this category. Most of the studies found to be using products’ images in different formats rather than the actual products as presented in Table 3. Yadava et al. [8] used images of 14 different products having 3 different varieties of each resulting in 42 product images for stimuli. Teo et al. [39] presented 60 different bracelet-like 3D shapes to the participants to record their EEG. Kumar et al. [9] used 14 online product categories with 3 variants for each category resulting in a total of 42 different product images. Goto et al. [70] recorded participants’ EEG while performing a virtual shopping task, and each participant was shown a series of 180 products, including electronics, food, beverages, and sports equipment. Gong et al. [74] used 480 product images from 4 categories: shampoo, water cup, headset, and USB flash drive. García-Madariaga et al. [79] used 63 product images from 3 categories: drinks, salty snacks, and sweet appetizers. Fu et al. [80] used colored images of 20 products with real and fake prices. Izadi et al. [90] used 40 images of sports products containing four strategies: advertising, discount, charity, and endorsement. Ma et al. [114] used different images consisting of product names and brand names. Özbeyaz [116] used 10 branded and 10 unbranded smartphone images as stimuli.

**Table 3:** Stimuli used in neuromarketing applications

Stimuli	Neuromarketing studies	Percentage of studies
Product	Products’ images	
	Yadava et al. [8], Hakim et al. [36], Teo et al. [39], Aldayel et al. [40], Aldayel et al. [42], Kumar et al. [9], Yen and Chiang [44], Zeng et al. [10], Raiesdana and Mousakhani [45], Kislov et al. [46], Al-Nafjan [47], Shah et al. [48], Hassani et al. [49], Georgiadis et al. [50], Göker [51], Shen et al. [52], Domracheva and Kulikova [57], Ramsøy et al. [59], Goto et al. [62], Eijlers et al. [63], Wang et al. [66], Ma et al. [67], Kakaria et al. [68], Goto et al. [70], Jin et al. [71], Çakar et al. [72], Gong et al. [74], Gholami Doborjeh et al. [76], García-Madariaga et al. [79], Fu et al. [80], Zubair et al. [85], Zhao and Wang [89], Izadi et al. [90], Wang et al. [93], Ma et al. [94], Yu et al. [95], Kim et al. [97], Wang et al. [98], Alvino et al. [99], Wang et al. [101], Hassani et al. [102], Wei et al. [103], Damião de Paula et al. [104], Ma et al. [114], Özbeyaz [116], Yang and Kim [117], Camarrone and Van Hulle [118]	54.02%
	Actual products	
	Li et al. [43], Lukovics et al. [69], Alvino et al. [83], Hsu and Chen [87], Pagan et al. [88], Robertson et al. [91], Mengual-Recuerda et al. [96], Hsu and Chen [110], Pennanen et al. [111]	10.34%
Promotion	Hakim et al. [37], Guixeres et al. [38], Oikonomou et al. [41], Wajid et al. [55], Gountas et al. [56], Wei et al. [58], Harris et al. [61], Vozzi et al. [64], Zito et al. [65], Royo et al. [73], Daugherty et al. [75], Gordon et al. [78], Golnar-Nik et al. [81], Hsu and Chen [86], Russo et al. [100], Russo et al. [105], Uhm et al. [113], Garczarek-Bąk et al. [115], Camarrone and Van Hulle [118]	21.84%
Others	Uhm et al. [53], González-Morales [54], Yang et al. [60], Alonso Dos Santos and Calabuig Moreno [77], Sänger [82], Verhulst et al. [84], Martinez-Levy et al. [92], Bosshard and Walla [106], Song et al. [107], Avinash et al. [108], Michael et al. [109], Leeuwis et al. [112]	13.79%

Some studies were found to use the actual products as stimuli as shown in Table 3. Li et al. [43] used different ladies’ shirts. Lukovics et al. [69] used real life experience of self-driving vehicles to record EEG. Alvino et al. [83] used 2 high price wines and 2 low price wines. Some studies used different snack products [87, 111]. Pagan et al. [88] and Hsu and Chen [110] used 2 wines from two different countries of origin. Robertson et al. [91] used 20 different white wine varieties to record EEG.

#### Promotion

The studies that used only video commercials are included in this category as presented in Table 3. Hakim et al. [37] used 3 video commercials for 6 food products. Gountas et al. [56] used five video commercials with alcohol reduction themes. Royo et al. [73] used a video commercial of a pushchair. Gordon et al. [78] used 4 narrative videos focused on important energy use habits, including refrigerators, lighting, laundry, and appliances. Golnar-Nik et al. [81] used video ads from four popular mobile brands (Samsung, Apple, Meizu, and Nokia). Hsu and Chen [86] used hotel videos with and without subtle effects. Russo et al. [100] used two video commercials of traditional cheese as stimuli to record EEG data.

#### Others

Some studies used normal pictures, affective images, music, movies, website visiting, and texts as stimuli and related them to Neuromarketing applications. These stimuli are taken into account in this category as shown in Table 3. González-Morales [54] and Martinez-Levy et al. [92] used different affective images. Alonso Dos Santos and Calabuig Moreno [77] used sponsored messages and montages. Sänger [82] used “food vs. neutral” pictures for participants to perform a central oddball task. Avinash et al. [108] used different types of classic music. Michael et al. [109] used multiple photos and videos of travel destinations.

**Fig. 4:**
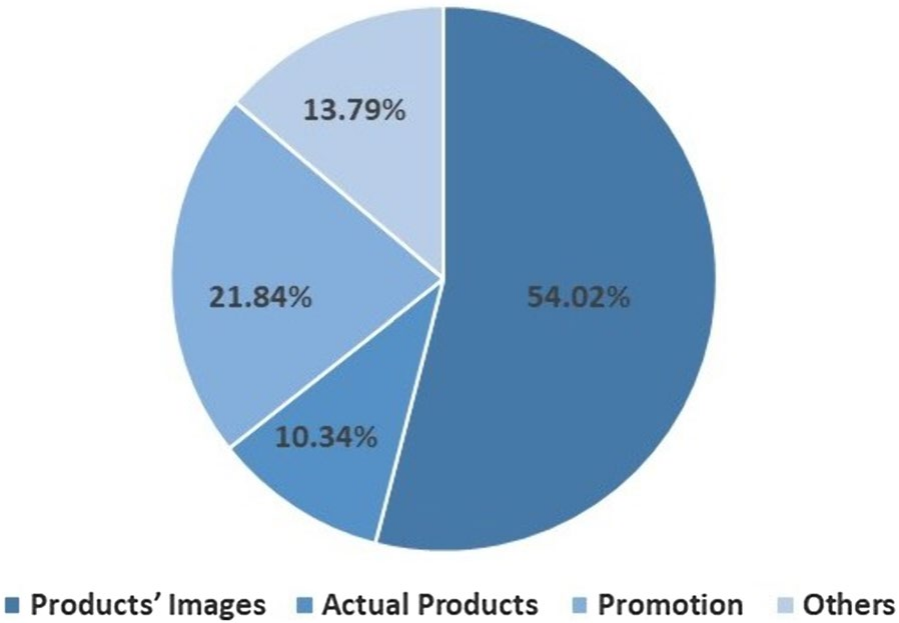
Stimuli used in Neuromarketing applications

A summary of the marketing stimuli used in the literature according to the three categories is shown in Fig. 4. It shows that the majority of the studies in the literature used products’ images as marketing stimuli to record the brain data. It is due to the popularity of online shopping where consumers typically view product images with accompanying details. Neuromarketing researchers should aim to create a realistic purchasing environment for consumers, regardless of the type of stimuli used. This will help to reveal the genuine thoughts of consumers when making purchasing decisions. However, the technology improvements have shifted the focus of marketing stimulus away from the TV commercials and toward the images of the products. While recording the data, the participants should not be given a time limit to observe the images, as this can cause pressure and does not reflect a natural purchasing environment.

Neuromarketing researchers should strive to create an authentic purchasing setting to reveal consumers' honest thoughts when making purchasing decisions, regardless of the stimuli employed. However, as technology advances, people increasingly use digital media and social networks to compare and shop for products rather than physically visiting stores. Therefore, future marketing research should focus on using images of products as stimuli to elicit genuine emotional responses through brain data rather than relying on traditional marketing methods such as TV commercials and physical product comparisons. When collecting data, creating a realistic buying scenario for participants is essential. Allowing them ample time to view products without any time constraints will provide a more accurate representation of the purchasing environment and prevent undue pressure on their decision-making process. Using a justified sample size is also critical to obtain statistically significant results. For stimuli around 15 s, the sample size should be a minimum of 32, and for stimuli around 30 s, it should be a minimum of 24, as found in the study of [64].

#### Available datasets

There are some publicly available Neuromarketing EEG datasets that researchers can use to jump into analysis quickly [8, 125, 126]. The dataset in [8] is one of the most commonly used public datasets for Neuromarketing research and was used in several studies [8, 40, 48, 51, 122, 127]. Twenty-five participants were involved in this study, where EEG data was recorded using the Emotiv EPOC + headset with a sampling frequency of 128 Hz. The subjects were exposed to 42 different product images while their EEG data were recorded. The EEG data were collected from 14 channels located at AF3, F7, F3, FC5, T7, P7, O1, O2, P8, T8, FC6, F4, F8, and AF4 locations on the head following international 10–20 system.

Georgiadis et al. [125] have provided another publicly available EEG dataset. They conducted a study with 42 participants who were presented with six supermarket brochures containing 144 products while their EEG and Eye-tracking (ET) data were recorded. They used Wearable Sensing's DSI 24 device to record EEG data and Tobii Pro Fusion to record ET data. The EEG data was collected from 21 channels located at Fp1, Fp2, Fz, F3, F4, F7, F8, Cz, C3, C4, T7/T3, T8/T4, Pz, P3, P4, P7/T5, P8/T6, O1, O2, A1 and A2 following the international 10–20 system at a sampling frequency of 300 Hz.

Mashrur et al. [126] presented another new EEG dataset for the prospective research community. They recorded EEG data from 20 participants while exposed to 5 different endorsement and promotion-based advertisements. The data was collected using an Emotiv EPOC + headset with a sampling frequency of 128 Hz. The EEG data was collected from AF3, AF4, F3, F4, F7 and F8 positions following the international 10–20 system.

### Pre-processing techniques in neuromarketing

EEG signals are very susceptible to noises, such as cardiac signals – Electrocardiogram (ECG), visual signals produced by eye movements—Electrooculogram (EOG), movement artifacts generated by muscle contractions—Electromyogram (EMG), and power line interference (50/60 Hz) caused by power line frequency. The pre-processing stage removes these noises and prepares the signal within the desired frequency band for further processing. Properly pre-processing the raw EEG data is crucial since noisy EEG data will likely not give better results.

EEG is affected by low-frequency noises caused by various sources such as eye movement, head, electrode wires, and sweat on the scalp. The low-frequency noise is characterized by slow drifts in the EEG signal over several seconds. On the other hand, high-frequency noise is caused by eye blinks and muscle contractions, particularly in facial and neck muscles. High-frequency noise manifests itself as quick up-down changes in the EEG signal.

The EEG signal has a frequency band ranging from around 0.5 to 100 Hz, leading to the common usage of Band Pass Filter (BPF) [42, 56, 73, 77, 92], Low Pass Filter (LPF) [52, 54, 80], and High Pass Filter (HPF) [72, 88, 89]. Notch Filter, which has response characteristics quite the opposite of the BPF, is often used to eliminate power line interference (50/60 Hz) [39, 73, 115]. Another filtering technique found in the literature is the Savitzky-Golay (S-Golay) filter, which is used to smooth the signal and increase the precision of the acquired data without changing the tendency of the raw data [8, 9, 42]. This is accomplished using the convolution technique, which involves utilizing linear least squares to fit successive subsets of neighboring data points with a low-degree polynomial [128].

Among the filters, some studies adopted Finite Impulse Response (FIR) filters [60, 117], while some others adopted Infinite Impulse Response (IIR) filters [61, 65]. The choice between FIR and IIR filters depends on the data being used and the desired application. For a sharp transition in the frequency domain and to prevent the creation of excess artifacts due to passband ripples, an IIR filter should be used [129]. The FIR filter requires a higher order than the IIR filter to achieve the same level of performance [130]. This means that the FIR filter requires more processing time than the IIR filter [130]. However, FIR filters are always stable regardless of the input signals.

Independent Component Analysis (ICA) is a blind source separation technique widely used as a pre-processing tool in Neuromarketing applications [37, 42, 54, 55, 81, 88, 92]. It is a valuable technique for analyzing multi-channel signals and separating the signal into its additive subcomponents. The noises, such as eye blink data, EMG, ECG, and EOG, are divided into different subcomponents, which can be removed to obtain the original data. Segmentation is also commonly used as a pre-processing technique to separate the signal into multiple epochs with identical statistical characteristics in terms of time and frequency [52, 55, 70, 71, 82, 83, 89, 108].

In the literature, most of the studies combined various abovementioned pre-processing techniques to create pipelines that take raw EEG data as input and produce noise-free EEG data as output. Automating pre-processing pipelines can save time and make them more efficient [131]. EEGLAB [132] was the most commonly used tool to prepare these pipelines [54, 76, 81, 88, 91, 92, 100, 111]. Selecting the right pre-processing pipeline is crucial for getting accurate results. Most studies in the literature used a traditional pre-processing pipeline [42, 55, 92, 100]. Also, an advanced automated pipeline has been introduced recently in the literature [131]. In one of our studies [133], we compared the performance of these two pipelines and proposed an optimal EEG pre-processing pipeline that outperformed the previous two methods.

**Fig. 5:**
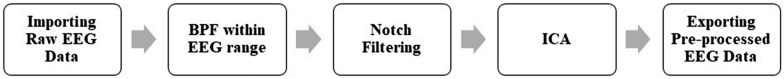
Traditional EEG pre-processing pipeline

The traditional pre-processing pipeline is presented in Fig. 5. This pipeline starts with bandpass filtering of the data within the desired EEG range followed by notch filtering (50 Hz/60 Hz) to remove the power line interferences, and ICA to remove eye and muscle noises.

**Fig. 6:**
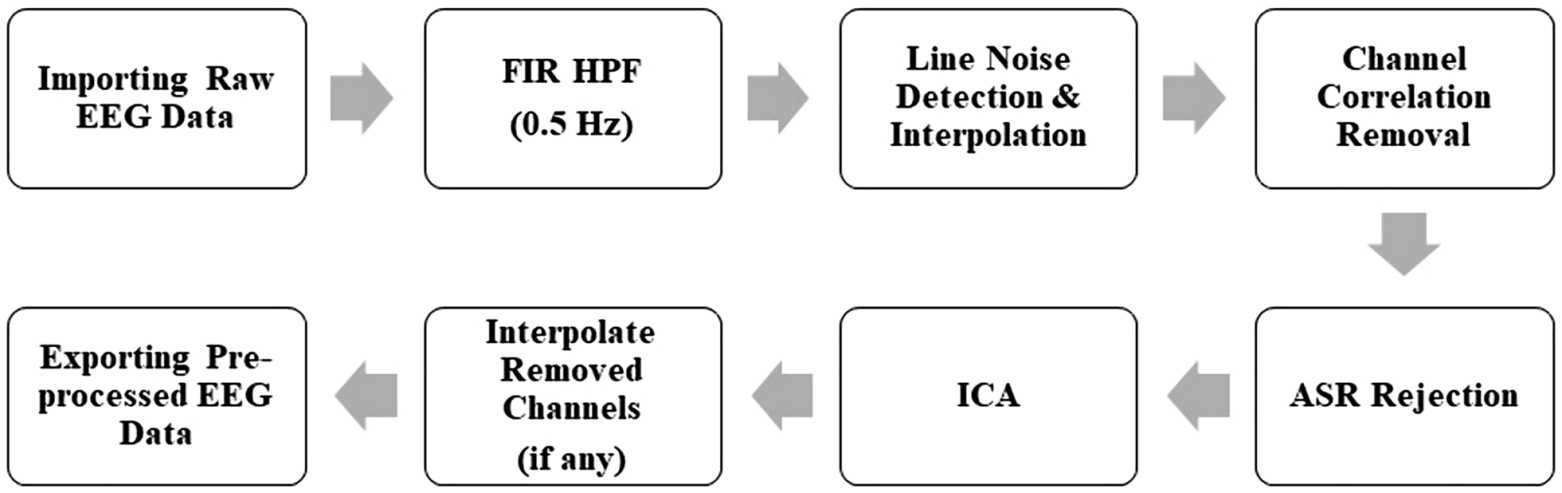
Automated EEG pre-processing pipeline

The traditional method treats a channel as a whole and cannot eliminate noisy data from specific parts. It cannot focus on a particular area of the channel. This limitation is addressed by the advanced automated EEG pre-processing pipeline which has been recently introduced in the literature [131]. This pipeline uses EEGLAB, as shown in the workflow diagram in Fig. 6, and resolves the issue by filtering out excessive noise from specific portions of the data.

**Fig. 7:**
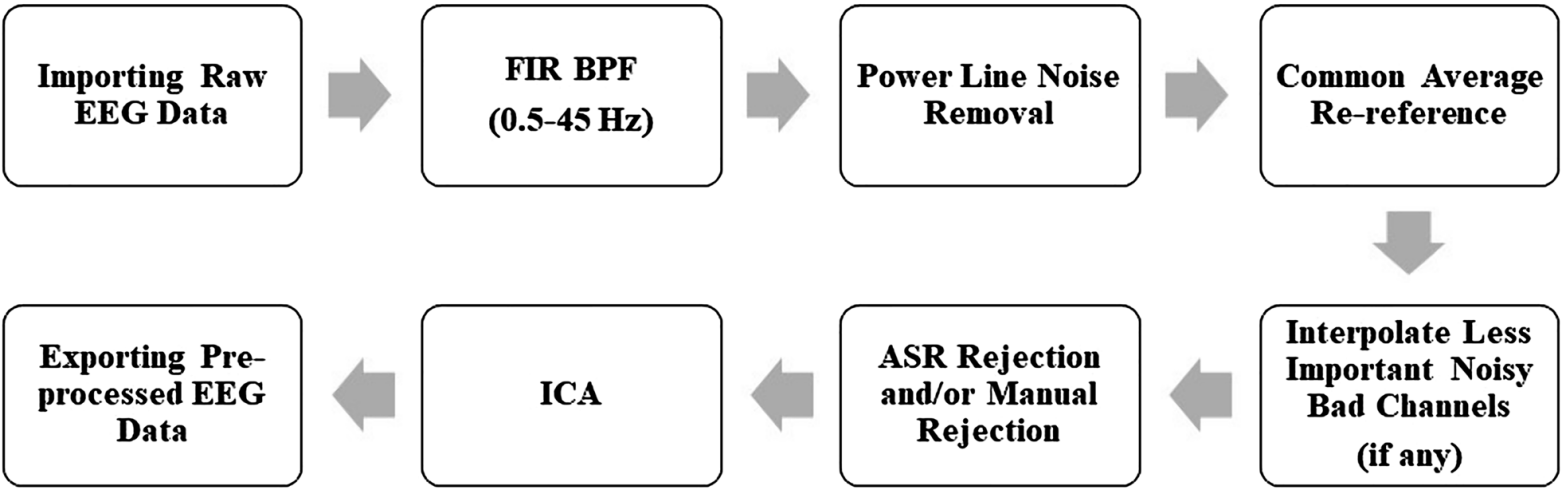
Optimal EEG pre-processing pipeline

The pre-processing pipeline starts with Finite Impulse Response (FIR) high pass filtering of the data at 0.5 Hz to remove any baseline drift. It then detects and interpolates any electrode line noise with a standard deviation (SD) threshold of 4, removes channel correlation with a threshold of 0.9, and applies Artifact Subspace Reconstruction (ASR) rejection with a threshold of 20. After that, it removes muscle and eye artifacts using ICA. Finally, any removed channels in the data are interpolated before exporting the pre-processed data. This automated EEG pre-processing pipeline is designed to first remove channels with high levels of noise and interpolate them later. However, this interpolation process can result in the loss of necessary information since an interpolated channel doesn't contain any original data. Its values are predicted from the potentials of the neighboring electrodes, which can compromise accuracy. For example, frontal electrodes are crucial in EEG-based preference prediction, as they help determine essential features such as Frontal Alpha Asymmetry (FAA) indexes. If the automated pipeline removes any of the frontal electrodes and then interpolates them, the corresponding indexes will not be accurate. Therefore, it is crucial to evaluate the results carefully and make necessary adjustments to ensure that the data is not compromised. We proposed an optimal EEG pre-processing pipeline that addresses these limitations and provides more accurate results [133]. In this pipeline, we used EEGLAB to process the EEG data. The workflow diagram is shown in Fig. 7.

First, we applied the FIR bandpass filter to the data within a range of 0.5–45 Hz and removed the power line noise (50 Hz) using the CleanLine plugin in EEGLAB [134]. This plugin uses multi-tapering and Thompson F-statistics to estimate and eliminate sinusoidal noises from the scalp channels, such as line noises [134]. Then, we re-referenced the data to the common average reference, addressing the re-referencing issue mentioned in [135]. We also checked whether any noisy bad channels are essential to the application of interest, and if not, we interpolated them subsequently. Next, we performed ASR rejection and/or manual rejection by examining the data. If the ASR rejection marked a large portion of the data for removal, we ignored it and performed manual rejection by removing only the significantly noisy part. Finally, we applied ICA to remove muscle and eye artefacts. Neuromarketing researchers can use this pipeline or other pipelines, but they should ensure that the pipeline they choose effectively cleans the raw data without losing necessary information.

EEG signals are susceptible to noises like cardiac, visual, muscle contraction, and power line interference. The pre-processing stage removes these noises and prepares the signal for further processing, ensuring better results. Studies have utilized various pre-processing techniques to create pipelines that convert raw EEG data into noise-free output, demonstrating the potential for automation to enhance efficiency. Neuromarketing researchers can use such pipelines, but they should ensure that the pipeline they choose effectively cleans the raw data without losing necessary information.

### Feature extraction techniques in neuromarketing

Feature extraction is simply the process of minimizing the number of resources needed to explain a significant amount of data. It is a technique for reducing the number of features by generating new ones from the old ones such that the new set of features can summarize the majority of the information present in the original feature set. Several different feature extraction approaches and features have been utilized in the literature as listed in Table 4.

**Table 4:** Feature extraction techniques used in neuromarketing

Feature extraction techniques	Neuromarketing studies
Discrete Wavelet Transform (DWT)	Yadava et al. [8], Aldayel et al. [40, 42], Kumar et al. [9], Al-Nafjan [47], Shah et al. [48], Hassani et al. [49, 102]
Power Spectra of EEG Sub-bands	Hakim et al. [36], Hakim et al. [37], Guixeres et al. [38], Teo et al. [39], Aldayel et al. [40], Oikonomou et al. [41], Aldayel et al. [42], Li et al. [43], Zeng et al. [10], Raiesdana and Mousakhani [45], Kislov et al. [46], Al-Nafjan [47], Shah et al. [48], Hassani et al. [49], Göker [51], Uhm et al. [53], González-Morales [54], Wajid et al. [55], Gountas et al. [56], Domracheva and Kulikova [57], Wei et al. [58], Ramsøy et al. [59], Harris et al. [61], Eijlers et al. [63], Vozzi et al. [64], Zito et al. [65], Lukovics et al. [69], Çakar et al. [72], Daugherty et al. [75], Alonso Dos Santos and Calabuig Moreno [77], Gordon et al. [78], Golnar-Nik et al. [81], Hsu and Chen [87], Pagan et al. [88], Robertson et al. [91], Martinez-Levy et al. [92], Kim et al. [97], Russo et al. [100], Hassani et al. [102], Damião de Paula et al. [104], Russo et al. [105], Avinash et al. [108], Pennanen et al. [111], Leeuwis et al. [112], Uhm et al. [113]
ERP	Shen et al. [52], Domracheva and Kulikova [57], Yang et al. [60], Goto et al. [62], Wang et al. [66], Ma et al. [67], Goto et al. [70], Jin et al. [71], Gong et al. [74], Daugherty et al. [75], Gholami Doborjeh et al. [76], Fu et al. [80], Sänger [82], Zubair et al. [85], Zhao and Wang [89], Wang et al. [93], Ma et al. [94], Yu et al. [95], Wang et al. [98], Wang et al. [101], Wei et al. [103], Bosshard and Walla [106], Song et al. [107], Ma et al. [114], Yang and Kim [117], Camarrone and Van Hulle [118]
EEG Asymmetry Indices	Hakim et al. [37], Guixeres et al. [38], Aldayel et al. [40], Oikonomou et al. [41], Zeng et al. [10], Kislov et al. [46], Vozzi et al. [64], Zito et al. [65], Kakaria et al. [68], Lukovics et al. [69], Çakar et al. [72], García-Madariaga et al. [79], Verhulst et al. [84], Martinez-Levy et al. [92], Mengual-Recuerda et al. [96], Russo et al. [100], Damião de Paula et al. [104], Russo et al. [105], Michael et al. [109], Garczarek-Bąk et al. [115]
Statistical Features	Yadava et al. [8], Aldayel et al. [40], Zeng et al. [10], Al-Nafjan [47], Hassani et al. [49], Wei et al. [58], Hassani et al. [102]

#### DWT

Many studies have been found in the literature that used DWT technique to extract wavelet coefficients as EEG features corresponding to different EEG frequency bands [8, 9, 40, 42, 47–49, 102]. The DWT is a signal analysis method that decomposes signals into different coefficients in the time–frequency domain. It can be described as a multi-resolution or multi-scale analysis where each coefficient provides a unique representation of the input signal. The DWT uses a convolution operation, which is a two-function multiplication process, to generate wavelet coefficients. The resulting inner product of each wavelet with the input signal produces a unique coefficient [40, 136, 137]. The DWT can be mathematically expressed using Eq. (1).

1 $$D\left(i,j\right)= \sum_{n=0}^{M-1}x\left(n\right).{\varphi }_{i,j}^{*}(n)$$

where $x\left(n\right)$ is a signal of length $n$, and ${\varphi }_{i,j}^{*}(n)$ is scaling wavelet function. In DWT decomposition, a filter bank consisting of a group of high- and low-pass filters is used. The low-pass filters generate approximation coefficients, while the high-pass filters produce wavelet detail coefficients. The accuracy of DWT analysis depends on the selection of wavelet technique and the number of wavelet decomposition levels [8, 40, 136, 137]. The number of wavelet decomposition levels depends on the sampling frequency of the signals. For 128 Hz sampling frequency, the four-level signal decomposition technique Daubechies 4 (DB4) was used to extract the wavelet coefficients A_4_, D_4_, D_3_, D_2_, D_1_ corresponding to the EEG frequency bands δ (0.5–4 Hz), θ (4–8 Hz), α (8–13 Hz), β (13–30 Hz), and γ (30 ~ Hz) [8, 9, 40].

#### Power spectra of EEG sub-bands

Power spectra is an indicator of power in a certain signal in terms of frequency [138]. It is the most commonly used feature in Neuromarketing studies as presented in Table 4. The asymmetry in EEG band power is an indicator of consumer preferences, which is why it is widely used in Neuromarketing [10, 40, 68, 72]. To determine the power spectra of EEG sub-bands, firstly a Fast Fourier Transform (FFT) algorithm is used to calculate the Discrete Fourier Transform (DFT) of the EEG signal sequence $x\left(n\right)$ as given by Eq. (2) [43, 53, 88, 108]. Such Fourier analysis is used to convert a signal from its original time domain to frequency domain. The DFT decomposes the signal sequence $x\left(n\right)$ into its components of different frequencies.

2 $${X}_{k}=\sum_{n=0}^{N-1}{x}_{n}.{e}^{-\frac{i2\pi kn}{N}}$$

where ${X}_{k}$ represents the frequency component at index *k*, ${x}_{n}$ is the input value at index *n*, and *N* is the data size. Then different periodogram techniques such as Welch’s Periodogram [139] have been used to calculate the power spectral density (PSD) of different EEG sub-bands as given by Eq. (3) [10, 40, 42, 45, 81, 91].

3 $${P}_{f}=\frac{1}{N}{\sum }_{n=0}^{N-1}{\left|{X}_{n}\left(k\right)\right|}^{2}$$

where ${P}_{f}$ presents the PSD of ${X}_{n}\left(k\right)$ corresponding to the *n*th segment and the *k*th frequency point after windowing. The relative power of different EEG frequency bands δ (0.5–4 Hz), θ (4–8 Hz), α (8–13 Hz), β (13–30 Hz), and γ (30 ~ Hz) are calculated this way, and extensively used as features in Neuromarketing applications. Some studies used Global Field Power (GFP) to get the spatiotemporal standard deviation of activity of each EEG time point [38, 64, 78, 92].

#### ERP

ERPs are voltage variations in EEG activity that correspond to motor, cognitive, and sensory events in real-time. They use the synchronized activity of neural populations to categorize and identify linguistic, memory, and perceptual processes. ERPs are used to extract event-related activity that is hard to discern from continuous EEG activity by averaging the electrocortical responses that occur throughout each event repetition [140]. Various Neuromarketing studies have linked ERPs to consumers' purchasing behavior, as shown in Table 4.

ERPs can generally be expressed as small spikes in brain activity that occur in response to a specific stimulus. These spikes have extremely low amplitudes, which makes it necessary to average EEG samples over many iterations to recognize ERPs and remove noise oscillations [122]. Table 5 presents the most common ERPs used in Neuromarketing research.

**Table 5:** ERPs used in neuromarketing

ERP	Brain Region	Interpretation	Neuromarketing studies
N2/N200	Central, frontal, and fronto-central regions	Indicates consumers’ conflicts during decision making process	Yang et al. [60], Goto et al. [62], Wang et al. [66], Goto et al. [70], Jin et al. [71], Gong et al. [74], Fu et al. [80], Wang et al. [93], Yu et al. [95], Yang and Kim [117]
P2/P200	Prefrontal region	Indicated positively correlation between consumers' attention and negative stimuli	Jin et al. [71], Gong et al. [74], Gholami Doborjeh et al. [76], Sänger [82], Zhao and Wang [89], Ma et al. [94], Wang et al. [98], Wei et al. [103]
P3/P300	Posterior region	Indicates both decision-making conflicts and the difficulty in reaching a decision	Gong et al. [74], Sänger [82], Zubair et al. [85]
Late Positive Potential (LPP)	Centro-parietal region	Indicates intense attention to significant stimuli	Shen et al. [52], Goto et al. [62], Goto et al. [70], Jin et al. [71], Fu et al. [80], Zhao and Wang [89], Wang et al. [93, 98, 101], Ma et al. [94, 141], Yu et al. [95], Wei et al. [103], Bosshard and Walla [106], Song et al. [107]
N400	Centro-parietal region	Indicates negative correlation between engagement and stimuli	Domracheva and Kulikova [57], Yang et al. [60], Song et al. [107], Camarrone and Van Hulle [118]
Positive Slow Waves (PSW)	Fronto-central and parietal regions	Indicates working memory functions	Goto et al. [62], Goto et al. [70]

##### N200

The N2/N200 is an ERP component that shows a negative voltage. It occurs around 200 ms after the stimulus onset and is mainly located in the central, frontal, and fronto-central areas of the brain [142]. N200 is an indicator of the decision maker’s conflict monitoring during the decision-making process [143]. Goto et al. [70] reported that observing consumer products that were highly preferred produced greater positive N200 amplitudes than watching products that were less preferred. Gong et al. [74] investigated the effect of different online sales promotions on perceived risk and found a positive correlation with N200 amplitude. Fu et al. [80] found that the truthful condition resulted in a weaker N2 response when compared to the deceptive condition. This indicates a lower cognitive and decisional conflict.

##### P200

P2/P200 is an ERP component that appears early in the decision-making process, approximately 200 ms after the stimulus onset. The prefrontal area contains the majority of this component [144]. Gong et al. [74] used the P200 component to demonstrate how consumers initially evaluate the effectiveness of the stimulus. They found that people pay more attention to negative stimuli, and there is a positive correlation between the subjects' attention to negative stimuli and the P200 amplitude. In certain situations, the components of P200 and N200 can overlap, creating a P200/N200 complex [71, 145]. To quantify the P200/N200 complex in such situations, peak-to-peak scores are utilized [62, 146]. Goto et al. [62] extracted the positive and negative peak amplitudes from two specific time windows, 130–230 ms for P200 and 200–400 ms for N200. The peak-to-peak measurements of N200 were computed for each electrode by subtracting the P200 peaks from the N200 peaks.

##### P300

The P3/P300 wave is a brain activity that peaks between 300 and 400 ms and has a generally positive voltage [147]. It can be observed in various areas of the brain, and larger amplitudes are usually found in the posterior brain [148]. P3 can indicate both decision-making conflicts and the difficulty in reaching a decision. Both N2 and P3 are related to conflict resolution and serve as indicators of the degree of conflict in decision-making processes [74, 149]. In addition, P3 also reflects the difficulty and confidence involved in making decisions [74]. As decision-making difficulty increases, the P3 amplitude decreases, while decision-making confidence correlates positively with the P3 amplitude [74, 150]. P300 also displays both attention activity in working memory and response adjustment [122]. Zubair et al. [85] observed a higher value of the P300 component in positive framing messages, indicating that non-threatening emotional information is processed with more attention.

##### LPP

The LPP is a positive deflection that is frequently observed between 400 and 800 ms, with its highest point generated from the centro-parietal region of the brain. It can be linked to attention toward emotional stimuli with positive or negative valence [122]. It indicates an intense attention to significant stimuli. This deflection is often observed in response to visual information that conveys emotional content [62, 151]. Ma et al. [141] reported that the larger LPP amplitudes for the prices of products were positively correlated with buying intentions. Fu et al. [80] discovered that the truthful condition elicited a greater LPP response than the deceptive condition. This suggests less cognitive and decisional conflict and a more positive evaluation of the truthful condition. The service with higher emotional arousal elicits a greater LPP amplitude compared to the service with lower emotional arousal [89].

##### N400

N400 is a negative deflection that reaches its maximum around 400 ms after the stimulus onset and is typically observed over the centro-parietal electrode sites [57]. Researchers use this deflection to study the effects of brand familiarity and brand extension services in Neuromarketing [60, 118]. It has been found that a large N400 response indicates low association strength in the customer's mind, and vice versa [118].

##### PSW

The PSW is a positive deflection that occurs after 800 ms and can last up to 3 s after a visual stimulus is presented [70]. It is commonly observed in both fronto-central and parietal sites and indicates a relatively prolonged form of processing that may involve working memory functions [62, 152]. Goto et al. [62] observed higher prediction accuracy rates with later positivities like the LPP and PSW compared to the N200 of the frontal area.

#### EEG asymmetry indices

Table 4 shows that many studies used brain activation asymmetry indices as features to predict consumer preferences. We have found four different types of asymmetry indices in the literature that can be used as autonomic indicator of consumers’ preferences—(1) Approach-Withdrawal (AW) Index; (2) Valence Index; (3) Effort Index; and (4) Choice Index.

##### AW index

The AW index is a measure of frontal alpha asymmetry, which indicates the difference in activations between the left and right hemispheres. It estimates desire and motivation by assessing alpha's higher activation in the right frontal cortex [40, 47, 59, 120, 122, 153]. Many studies have exhibited the effectiveness and accuracy of FAA as a crucial factor in emotion and Neuromarketing research [10, 47, 59, 65, 92, 100, 105, 120, 124]. To calculate the AW index, we can use electrodes F4 and F3 to determine the difference between the right and left PSD using either Eq. (4) [47, 153] or Eq. (5) [47, 120].

4 $$A{W}_{1}= \alpha \left(F4\right)-\alpha \left(F3\right)$$

5 $$A{W}_{2}= \frac{\alpha \left(F4\right)-\alpha (F3)}{\alpha \left(F4\right)+\alpha (F3)}$$

##### Valence index

Studies have found a link between frontal asymmetry and a customer’s emotional state [10, 47, 59, 65, 92, 100, 120, 124]. Specifically, left frontal activation is associated with positive valence, while right frontal activation is associated with negative valence. Several studies have supported the theory that frontal EEG asymmetry is an indicator of valence [10, 40, 47, 122]. It can be calculated by either of the Eqs. (6) [47, 124], (7) [47, 124], or (8) [10, 154].

6 $$Valenc{e}_{1}= \frac{\beta \left(F3\right)}{\alpha \left(F3\right)}-\frac{\beta \left(F4\right)}{\alpha \left(F4\right)}$$

7 $$Valenc{e}_{2}=\text{ln}\left[\alpha \left(F3\right)\right]-\text{ln}\left[\alpha \left(F4\right)\right]$$

8 $$Valenc{e}_{3}= \frac{\alpha \left(F4\right)}{\beta \left(F4\right)}-\frac{\alpha \left(F3\right)}{\beta \left(F3\right)}$$

##### Choice index

The choice index is a measure based on the frontal asymmetric gamma and beta oscillations, which are primarily associated with the actual decision-making process. It is also highly correlated with willingness-to-pay responses, particularly in the gamma band, which is used to assess consumer preference and choice [59]. Greater activation in the left prefrontal region is indicated by higher values in the gamma and beta bands, whereas the right prefrontal region is associated with considerably stronger activation at lower levels [10, 47, 59]. Choice index using gamma band and beta band can be expressed by Eq. (9) and (10) [10, 59].

9 $$Choice \,Inde{x}_{\gamma }= \frac{\text{log}\left(\gamma \left(Electrod{e}_{left}\right)\right)-\text{log}\left(\gamma \left(Electrod{e}_{right}\right)\right)}{\text{log}\left(\gamma \left(Electrod{e}_{left}\right)\right)+\text{log}\left(\gamma \left(Electrod{e}_{right}\right)\right)}$$

10 $$Choice \,Inde{x}_{\beta }= \frac{\text{log}\left(\beta \left(Electrod{e}_{left}\right)\right)-\text{log}\left(\beta \left(Electrod{e}_{right}\right)\right)}{\text{log}\left(\beta \left(Electrod{e}_{left}\right)\right)+\text{log}\left(\beta \left(Electrod{e}_{right}\right)\right)}$$

##### Effort index

The activity level of the frontal theta in the prefrontal cortex is a measure that indicates the cognitive processing that arises from mental exhaustion. Higher levels of theta activity are associated with harder and more complicated tasks in the frontal area. This measure has been frequently studied in Neuromarketing research [81, 108, 123, 155–157]. We can use Eq. (11) to represent the effort index [108].

11 $$Effort \,Index= \frac{10\text{ log}\left(\theta \left(Electrod{e}_{left}\right)\right)-\text{log}\left(\theta \left(Electrod{e}_{right}\right)\right)}{\text{log}\left(\theta \left(Electrod{e}_{left}\right)\right)+\text{log}\left(\theta \left(Electrod{e}_{right}\right)\right)}$$

#### Statistical features

Different types of statistical features have been utilized in the literature as listed in Table 4. Mostly used statistical features are—Mean, SD, Variance, Skewness, Kurtosis, and Differential Entropy (DE). Apart from these features, some studies also used Hjorth parameters as features in Neuromarketing [10, 158]. To analyze the EEG signal in the time domain, Hjorth developed the Hjorth parameters [159]. These parameters consist of three measures: activity, mobility, and complexity. Activity measures signal amplitude deviation, mobility measures slope changes, and complexity measures amplitude standard slope count [10].

Feature extraction techniques reduce the resources needed to explain large amounts of data by generating new features that summarize most of the original set. Various feature extraction techniques have been used in the literature to extract different time, frequency, and time–frequency domain features. As already shown in the systematic review, these features correspond to different interpretations related to consumers' decision-making process. Proper features should be selected based on the study's objectives to interpret the data better.

### Data interpretation techniques in neuromarketing

The studies used either machine learning or statistical analysis to interpret data. They employed advanced machine learning algorithms to classify consumers' like/dislike decisions. On the other hand, various statistical analysis techniques were used for correlational and behavioral analyses. It is crucial to select appropriate ML or statistical analysis techniques to accurately interpret the data.

#### Machine learning applications in neuromarketing

The Neuromarketing experiments utilized both supervised and unsupervised learning methods. In supervised learning, a priori ground truth, which is usually the interviewed response of the test subjects (like/dislike), is used as the labels. In the training datasets, these labels aid the classifier in recognizing the signal sequence of like and dislike. In the testing stage, a label-free dataset is used to predict the like/dislike responses. On the contrary, previous knowledge of the like/dislike labeling is not necessary for the unsupervised learning methods. The most commonly used machine learning methods are listed in Table 6. Supervised learning methods include the Hidden Markov Model (HMM), Support Vector Machine (SVM), k-Nearest Neighbors (k-NN), Neural Network (NN), Deep Learning (DL), Random Forest (RF), Regression, and Linear Discriminant Analysis (LDA).

**Table 6:** Machine learning techniques used in neuromarketing

Machine learning techniques	Neuromarketing Studies	Average classification accuracy
HMM	Yadava et al. [8]	70.33% (male) and 63.56% (female) [8]
SVM	Hakim et al. [37], Aldayel et al. [40, 42], Li et al. [43], Zeng et al. [10], Georgiadis et al. [50], Wei et al. [58], Gholami Doborjeh et al. [76], Golnar-Nik et al. [81], Yang and Kim [117]	80.28% [43], 75% [58], 87% [81]
k-NN	Hakim et al. [37], Aldayel et al. [40, 42], Zeng et al. [10], Raiesdana and Mousakhani [45], Georgiadis et al. [50], Avinash et al. [108]	94.22% [10], 92.4% [45], 82.75% [108]
NN	Guixeres et al. [38], Gholami Doborjeh et al. [76], Özbeyaz [116]	82.9% [38], 90% [76], 72% [116]
DL	Hakim et al. [36], Teo et al. [39], Aldayel et al. [40, 122], Al-Nafjan [47], Göker [51], Georgiadis et al. [160], Alimardani and Kaba [161]	75.09% [36], 79.76% [39], 93% [40], 99% [47], 96.83% [51], 72.18% [160], 74.57% [161]
RF	Aldayel et al. [40, 42], Kumar et al. [9], Al-Nafjan [47], Hassani et al. [49, 102]	100% [47], (71 .51 ± 5 .1%) [49], 96.47% (female) & 95.32% (male) [102]
Regression	Hakim et al. [37], Kislov et al. [46], Gholami Doborjeh et al. [76], Izadi et al. [90], Garczarek-Bąk et al. [115]	61.2% [115]
LDA	Golnar-Nik et al. [81], Avinash et al. [108]	87% [81], 90% [108]

In supervised learning techniques, the HMM is a non-linear classifier that is commonly used in biomedical and spatial signals. It originated from statistical modeling and helps classify multiple classes of consecutive data in Neuromarketing research. The HMM helps researchers identify potential states for observation by using state transition probabilities when there may be a transition from one mental state to another [1]. Yadava et al. [8] proposed an HMM-based consumer preference prediction model that outperformed other common classifiers including SVM, RF, and ANN. The model achieved a classification accuracy of 70.33% for male participants and 63.56% for female participants.

SVM is another supervised machine learning algorithm that uses training data to recognize patterns and derive relationships. SVM classifies by creating a hyperplane that separates different classes. The hyperplane is based on the training data and is used to classify new data. The accuracy and simplicity of computation of SVM make it a useful tool in Neuromarketing [1]. Li et al. [43] combined EEG and ET data for product design evaluation and obtained average classification accuracy of 80.28% using SVM classifier. Numerous studies employ LDA classifiers in contrast to SVM classifiers [81, 108]. LDA organizes several data points with comparable frequencies into discrete classes, and 1D Eigen transformation establishes these classes [1]. Golnar-Nik et al. [81] used SVM along with LDA to investigate the effects of changes in promotions’ contents on consumers’ minds and achieved an average classification accuracy of 87%.

Another supervised learning model is k-NN which can act as a regression and classification tool. Based on the *K* training samples that are the test sample's closest neighbors, the k-NN algorithm predicts the category of the test sample. Unlike SVM's hyperplane, k-NN establishes a decision boundary between several separate classes [1]. Using k-NN algorithm for the preference classification tasks, Raiesdana and Mousakhani [45] obtained an average classification accuracy of 92.4% for electric cars, and Zeng et al. [10] obtained 94.22% for sport shoes.

Different NN techniques have been utilized in the literature for classification purposes. The most commonly used NN techniques are Spiking Neural Network (SNN) [76], Artificial Neural Network (ANN) [116], and Multi-layer Perceptron (MLP) [76]. They use neurons or distributed nodes arranged in a stacked framework to mimic the structure of the human brain. They generate non-linear boundaries to make decisions across vast data sets. Computers can utilize this adaptive approach to learn from their errors and keep getting better. Even though they are becoming popular for data interpretation in Neuromarketing, they require a high number of features and data samples [1]. Guixeres et al. [38] explored whether NN models could be used to forecast the effectiveness of a new advertisement on digital channels, and the study's average classification accuracy was 82.9%.

DL models are best suited for predicting outcomes when there is a large dataset to learn from. Hakim et al. [36], Teo et al. [39], Aldayel et al. [40, 122], Al-Nafjan [47], Göker [51], Georgiadis et al. [160], and Alimardani and Kaba [161] used different DL models for the preference prediction tasks. Using DL models, Hakim et al. [36], Teo et al. [39], and Göker [51] achieved an average classification accuracy of 75.09% [36], 79.76% [39], and 96.83% [51], respectively. Using different feature selection algorithms along with the DL classifier Al-Nafjan [47] could achieve an average classification accuracy of 99%. Georgiadis et al. [160] proposed a decoding method using Riemannian Geometry principles and SPDNet deep learning architecture, which achieved an average classification accuracy of 72.18%. Alimardani and Kaba [161] used Convolutional Neural Network (CNN) as a deep learning model, obtaining an average classification accuracy of 74.57%.

A popular ensemble machine learning approach called random forest aggregates the output of several decision trees to produce a single outcome. Its versatility and ease of use, combined with its ability to address both regression and classification problems, have driven its popularity in Neuromarketing applications. When examining how color affects consumer choices, Hassani et al. [102] employed the RF algorithm to predict preferences. For female participants, this resulted in an average classification accuracy of 96.47%, whereas for male participants, it was 95.32%. Al-Nafjan [47] used RF algorithm with optimal features and obtained an average classification accuracy of 100%.

Selecting appropriate ML technique is essential to interpret the data accurately. Advanced ML algorithms were utilized to classify consumer preferences. Among the ML classifiers, ensemble learning-based models performed better in the literature. However, when selecting an ML algorithm, three things should be considered: the type of problem being addressed (linear or non-linear), the size of the data, and the number of features. For instance, simple classifiers such as SVM should be adopted for smaller sample sizes instead of complex NN classifiers.

#### Statistical analysis in neuromarketing

Biostatistics offers a range of statistical methods to analyze and interpret data for specific situations. It's essential to understand the assumptions and conditions of the statistical techniques to select the appropriate method for data analysis. The two main statistical methods are descriptive statistics and inferential statistics. Descriptive statistics summarize data using indexes such as mean, median, and standard deviation. Inferential statistics draw conclusions from the data using statistical tests such as t-test, Analysis of Variance (ANOVA) test, etc. Parametric and nonparametric are the two possible classifications for inferential statistical techniques. Statistical methods used for comparing means are referred to as parametric. In contrast, those used for comparing other variables like median, mean ranks, or proportions are referred to as non-parametric methods [162]. In the literature, a range of statistical analysis techniques have been employed for conducting correlational and behavioral analyses. The most commonly used statistical analysis techniques in Neuromarketing are summarized in Table 7.

**Table 7:** Statistical analyzing techniques used in Neuromarketing

Statistical analyzing techniques	Description	Neuromarketing studies
t-test	To compare the means of two paired groups (paired samples t-test) or unpaired groups (independent samples t-test)	Zeng et al. [10], Raiesdana and Mousakhani [45], Georgiadis et al. [50], Uhm et al. [53], Wajid et al. [55], Gountas et al. [56], Ramsøy et al. [59], Yang et al. [60], Harris et al. [61], Eijlers et al. [63], Jin et al. [71], Çakar et al. [72], Gong et al. [74], Verhulst et al. [84], Hsu and Chen [86], Hsu and Chen [87], Martinez-Levy et al. [92], Ma et al. [94], Yu et al. [95], Hsu and Chen [110], Pennanen et al. [111], Yang and Kim [117]
ANOVA	To compare the means of three or more unpaired (one-way ANOVA) or paired groups (repeated-measure ANOVA)	Guixeres et al. [38], Oikonomou et al. [41], Raiesdana and Mousakhani [45], Shen et al. [52], Uhm et al. [53], Ramsøy et al. [59], Yang et al. [60], Harris et al. [61], Goto et al. [62], Vozzi et al. [64], Zito et al. [65], Wang et al. [66], Ma et al. [67], Goto et al. [70], Jin et al. [71], Royo et al. [73], Gordon et al. [78], García-Madariaga et al. [79], Fu et al. [80], Sänger [82], Alvino et al. [83], Verhulst et al. [84], Zubair et al. [85], Zhao and Wang [89], Wang et al. [93], Ma et al. [94], Yu et al. [95], Kim et al. [97], Wang et al. [98], Alvino et al. [99], Russo et al. [100], Paula et al. [104], Bosshard and Walla [106], Song et al. [107], Pennanen et al. [111], Ma et al. [114]
Chi-square test	To compare the associations between two or more independent groups	Alonso Dos Santos and Calabuig Moreno [77], Hsu and Chen [86, 110], Garczarek-Bąk et al. [115]
Friedman test	To compare the medians/ interquartile ranges of three or more paired groups	Alvino et al. [83], Izadi et al. [90]
Wilcoxon Rank test	To compare the medians/ interquartile ranges of two paired (signed-rank test) or unpaired groups (sum test)	Domracheva and Kulikova [57], Kakaria et al. [68], Alonso Dos Santos and Calabuig Moreno [77]
Mann Whitney test	To compare the medians/ interquartile ranges of two unpaired groups	Domracheva and Kulikova [57], Pagan et al. [88], Garczarek-Bąk et al. [115]
Kruskal–Wallis test	To compare the medians/ interquartile ranges of three or more unpaired groups	Alonso Dos Santos and Calabuig Moreno [77]
Levene’s and Shapiro–Wilk’s test	To compare the variances between samples to verify whether or not a sample fits a normal distribution	Guixeres et al. [38], Alonso Dos Santos and Calabuig Moreno [77], Damião de Paula et al. [104], Russo et al. [105], Leeuwis et al. [112]
Descriptive statistics	To interpret data using indexes such as mean, median, and SD	Uhm et al. [53], González-Morales [54], Hsu and Chen [87]

The most frequently utilized parametric inferential statistical analysis methods are the t-test and ANOVA. The t-test is used to compare the means of two paired or unpaired groups. When it is used to compare two paired groups, it is called a paired samples t-test; when it is used to compare two unpaired groups, it is called an independent samples t-test. Zeng et al. [10] used two-sample t-tests to determine the difference in the EEG's power between the consumers' like and dislike decisions. Wajid et al. [55] employed the mean t-tests to investigate the variations in the message appeals of two ads. Çakar et al. [72] performed a t-test analysis on all the FAA-based data points to mark the values at a 95% significance level. The ANOVA is used to compare the means of three or more paired or unpaired groups. When it is used to compare paired groups, it is called one-way ANOVA; when it is used to compare unpaired groups, it is called repeated-measure ANOVA. Oikonomou et al. [41] performed a one-way ANOVA test to examine the impact of the classification techniques on accuracy values. Goto et al. [70] used repeated-measure ANOVA to investigate how the ERP waveforms were affected by subsequent purchasing decisions. Zubair et al. [85] used both one-way ANOVA and repeated-measure ANOVA on the ERP components to observe the effects of three different message-framing contents on consumers’ minds.

The most common nonparametric inferential statistical analysis methods are Chi-square test, Friedman test, Wilcoxon Rank test, Mann Whitney test, Kruskal–Wallis test, and Levene’s and Shapiro–Wilk’s test. Among them, the Chi-square test compares the proportions between different groups. In contrast, the other methods compare the medians/ interquartile ranges of different group patterns, as listed in Table 7. Kakaria et al. [68] observed the variations in cognitive stress during planned and unplanned purchases using the Wilcoxon Signed-Ranks test. Pagan et al. [88] divided study participants into two groups, recorded their EEG data, and performed the Mann–Whitney test to compare brainwave differences. Izadi et al. [90] employed the Friedman test to evaluate the difference in subjects’ alpha wave amount when they were exposed to different promotions. Garczarek-Bąk et al. [115] evaluated the predictive power of their model for private label purchasing using the Chi-square test. Different studies utilized Levene’s and Shapiro–Wilk’s tests to verify whether or not their samples fit normal distributions [104, 112]. Apart from these, descriptive statistics such as mean, median, and standard deviation were used by some studies to interpret the data [53, 54, 87].

Statistical techniques were employed for correlational and behavioural analysis. Biostatistics provides various statistical methods for data analysis, but understanding the assumptions and conditions of these techniques is crucial for selecting the most suitable method. Descriptive statistics are used to interpret the data using indexes (mean, median, and SD), and inferential statistics are employed to draw conclusions from the data using statistical tests (t-test, ANOVA test, etc.). When selecting the most appropriate statistical method, three key factors must be considered: the study's objective, the type and distribution of the data being analyzed, and the nature of the observations (paired or unpaired).

## Conclusions

Neuromarketing interprets customer preferences for goods and services using neuroscience, helping marketers avoid incorrect strategies. In this study, we reviewed recent publications to highlight research trends, technical scopes, and potential opportunities. We selected recent research articles from reliable databases that directly relate to experimental findings in Neuromarketing. Our findings indicate that the trend in Neuromarketing research has shifted from predicting consumer preferences to developing marketing strategies. We found that the medial-frontal brain region is particularly relevant when it comes to understanding consumers’ purchasing behavior. Furthermore, the focus of marketing stimuli has shifted from actual products and television ads to images of products on digital and social media. We also found that various pre-processing techniques can help remove noise from raw data, but it is essential to ensure that necessary information is not lost during this stage. Various feature extraction methods have been found in the literature extracting different time, frequency, and time–frequency domain features corresponding to varying interpretations of consumers’ decision-making processes. We presented that the selection of the appropriate features depends on the study’s objectives. Most studies were found to use ML algorithms or statistical analysis techniques to analyze consumer behavior. Among ML techniques, the ensemble learning-based models performed better than the others, and ANOVA is the most widely used technique among statistical analysis methods. However, we concluded that the choice of ML algorithm depends on the type of problem, the size of the data, and the number of features, while the selection of the appropriate statistical analysis method depends on the study's objective, the type and distribution of the data, and the nature of the observations. We hope that the findings and recommendations of this review will help future researchers find appropriate research direction and make novel contributions in this field.

## Data Availability

This review used available literature related to the problem statement from valid databases across the internet. Databases are Springer, Science Direct, IEEE, PubMed, and Emerald Insight.

## Abbreviations

BCI: Brain-computer interface
EEG: Electroencephalography
fMRI: Functional magnetic resonance imaging
MEG: Magnetoencephalography
PET: Positron emission tomography
fNIRS: Functional near-infrared spectroscopy
ML: Machine learning
ERP: Event related potential
ISC: Inter-subject correlations
PRISMA: Preferred Reporting Items for Systematic Reviews and Meta-Analyses
AI: Artificial intelligence
ECG: Electrocardiogram
EOG: Electrooculogram
EMG: Electromyogram
BPF: Band Pass Filter
LPF: Low Pass Filter
HPF: High Pass Filter
S-Golay: Savitzky-Golay
ICA: Independent component analysis
FIR: Finite impulse response
SD: Standard deviation
ASR: Artifact subspace reconstruction
FAA: Frontal alpha asymmetry
DWT: Discrete wavelet transform
FFT: Fast Fourier Transform
DFT: Discrete Fourier Transform
PSD: Power spectral density
GFP: Global field power
LPP: Late positive potential
PSW: Positive slow waves
AW: Approach-withdrawal
DE: Differential entropy
HMM: Hidden Markov Model
SVM: Support vector machine
NN: Neural Network
DL: Deep Learning
RF: Random forest
LDA: Linear Discriminant Analysis
k-NN: K-Nearest Neighbors
ET: Eye-tracking
SNN: Spiking Neural Network
ANN: Artificial Neural Network
MLP: Multi-layer Perceptron
CNN: Convolutional Neural Network
